# Estimation of Impacts of Global Factors on World Food Prices: A Comparison of Machine Learning Algorithms and Time Series Econometric Models

**DOI:** 10.3390/foods12040873

**Published:** 2023-02-18

**Authors:** Talat Ulussever, Hasan Murat Ertuğrul, Serpil Kılıç Depren, Mustafa Tevfik Kartal, Özer Depren

**Affiliations:** 1 Department of Economics and Finance, Gulf University for Science and Technology, Hawally 32093, Kuwait; 2 Center for Sustainable Energy and Economic Development (SEED), Gulf University for Science and Technology, Hawally 32093, Kuwait; 3 Department of Economics, Anadolu University, 26470 Eskişehir, Turkey; 4 Department of Statistics, Yildiz Technical University, 34220 İstanbul, Turkey; 5 Strategic Planning, Financial Reporting, and Investor Relations Directorate, Borsa Istanbul, 34467 İstanbul, Turkey; 6 Customer Experience Research Lab., Yapı Kredi Bank, 34330 İstanbul, Turkey

**Keywords:** global food prices, machine learning algorithms, time series econometric models, C13, C22, Q18

## Abstract

It is a well-felt recent phenomenal fact that global food prices have dramatically increased and attracted attention from practitioners and researchers. In line with this attraction, this study uncovers the impact of global factors on predicting food prices in an empirical comparison by using machine learning algorithms and time series econometric models. Covering eight global explanatory variables and monthly data from January 1991 to May 2021, the results show that machine learning algorithms reveal a better performance than time series econometric models while Multi-layer Perceptron is defined as the best machine learning algorithm among alternatives. Furthermore, the one-month lagged global food prices are found to be the most significant factor on the global food prices followed by raw material prices, fertilizer prices, and oil prices, respectively. Thus, the results highlight the effects of fluctuations in the global variables on global food prices. Additionally, policy implications are discussed.

## 1. Introduction

Needless to say, all countries aim not just to provide an economically and socially better lifestyle and living conditions to their citizens but also to improve the aforementioned conditions over the years. To do so, countries strive to achieve sustainable economic performance through economic growth and development [1]. It can be said that the economic performance of counties has a direct impact on the life quality of their citizens. Therefore, countries deal with supervising strictly main macroeconomic indicators, such as foreign exchange (FX) rates, gross domestic product (GDP), inflation, and stock markets [2]. Such indicators reflect how the well-being of the societies progresses and whether economies are sound and less risky concerning previous periods or their peers [3].

The indicators mentioned above have a wide range of associations with economic development, societies’ welfare, and citizens’ life quality [4]. Although developed and emerging economies try to improve their existing economic conditions, some emerging and frontier economies, unfortunately, try to deal with providing just basic needs to their citizens. In this respect, food is considered one of the most basic, significant, and critical needs for people. Although the progress of the economic indicators is important, it comes after meeting basic needs such as food.

Food, the most basic need, cannot be evaluated as a standard commodity and does not have any substitutes. People need food to sustain their lives and provide energy for their activities. Thus, it is necessary to have enough basic food continuously. However, it is not possible to store food in the human body. In the case of food deficiency, it is most likely that the person will encounter some diseases and it can even be fatal for people if food deficiency continues for a long time [5]. Although some other needs can be temporarily postponed, a postponement cannot be mentioned for the consumption of food [6].

It is obvious that food accessibility is significantly important for people, and becomes more critical, especially in the case of an increasing population. In such circumstances, not just accessibility but also the affordability of food becomes a high priority for both people and governments. Food accessibility is important since it affects the life quality of citizens. From the accessibility perspective, the progress of food prices has a vital role. That is why accessibility of people is exceedingly related to the price level.

Safe and sustainable means of food production are not enough to satisfy the food necessities of people. Physical and economical accessibility of food to citizens is also important. Accessibility to food includes affordability which depends on the product price as well. In addition to the main functions of food for people, food prices are also crucial from a macroeconomic perspective. Recently, increasing food prices have been the fundamental source of general price level changes. Specifically, an increase in food prices directly causes an increase in the inflation baskets (e.g., Consumer Price Index—CPI). Food prices have a high share in CPI baskets, especially in developing countries. For example, the share of the food prices in the CPI baskets is 21%, 26%, 28%, 30%, 33%, 39%, 40%, 42%, 50%, 51%, 62%, and 65% in South Africa, Turkey, China, Peru, India, Guatemala, Jordan, Egypt, Haiti, Kenya, Sri Lanka, and Bangladesh, respectively [7,8]. Therefore, distortions in food prices can negatively affect the price stability and purchasing power of the citizens. Moreover, the progress of food prices can affect macroeconomic indicators through the inflation channel. Thus, it can be stated that food prices are not only important for societies and people but also significant for countries and economies.

When examining the progress of global food prices, it can be seen that food prices have a relatively horizontal trend between 1991 and 2007. However, starting in 2007, food prices began to increase rapidly and have followed a highly fluctuating trend since then. Moreover, food prices have started to rise again with the occurrence of the COVID-19 pandemic [9].

Starting with the 2007–2008 food crisis, many studies have tried to address the causes, reasons, origins, and impacts of high and volatile global food prices and propose remedies. The global food price problem is, however, far-reaching, and its impacts on countries and people are widespread, significant, and interrelated.

In the current literature, the effects of energy prices, FX rates, climate change, and global warming on global food prices have been examined [10,11]. Therefore, the inclusion of various explanatory factors is required for the empirical examination of food prices. Moreover, focusing on global factors, such as energy (oil) prices, raw material prices, and fertilizer prices, can be a highly right approach while working on global food prices.

With the increase of food prices globally and its importance on countries, economies, societies, and people, this study examines the impact of global factors on global food prices through a comparative and comprehensive approach by employing both machine learning algorithms and time series econometric models. More specifically, we used eight global explanatory variables, which represent the supply side, and monthly data from January 1991 to May 2021. The outcomes of the empirical analysis mainly uncover that machine learning algorithms outperform time series econometric models and one-month-lagged global food prices are the most influential factors on global food prices followed by raw material prices, fertilizer prices, and oil prices, in order. Moreover, some policy proposals are recommended by considering the results obtained. Hence, the study aims to present policy implications to international institutions and governments of countries for managing food prices effectively to keep them under control.

The study contributes to the literature in several ways; (i) the study focuses on the estimation of global food prices by considering the impacts of global factors, which is not much studied; (ii) the study includes relatively long-term data from January 1991 to May 2021; (iii) the study employs both machine learning algorithms and time series econometric models to define the best estimation model by comparing estimation performance of the alternative models. According to our best knowledge, this is a leading study since there is no other study in the literature that performs both machine learning algorithms and time series econometric models to examine global food prices.

The remainder of this study is structured as follows. Section 2 reviews the literature. Section 3 details the methods. Section 4 presents the results of the empirical analysis including the comparison of the models applied and presenting the importance of variables obtained from the best model and discusses the results with recommending policy implications. The conclusion is given in Section 5.

## 2. Literature Review

One of the important factors for the progress of global food prices is economic policy uncertainty. While economic uncertainties, either global or national, are rising in an environment, adverse effects would inevitably occur. From the food price perspective, it can be expected that global food prices would surely surge while economic policy uncertainty intensifies. The correlation between economic policy uncertainty and food prices is examined in the literature. For example, Refs. [12,13] investigate the relationship between economic policy uncertainty and food prices by applying Nonlinear Autoregressive Distributed Lag (NARDL) and Vector Autoregression (VAR) approaches, and define that an increase in economic policy uncertainty negatively affects prices significantly. Thus, a positive (increasing) relationship between economic policy uncertainty and global food prices is expected in line with the related literature. Moreover, US Economic Policy Uncertainty Index is used as an economic policy uncertainty indicator in the empirical modeling.

Another important factor for global food prices is energy prices. Energy prices have a direct effect on food prices since they cause an increase in the production cost of food. In the literature, various studies e.g., Refs. [13,14,15,16,17,18,19,20] examine the relationship between energy prices and food prices by applying Dynamic Conditional Correlation (DCC), NARDL, and panel VAR models for Asian countries, China, United States (US). These studies mainly argue that adverse developments in energy prices, where oil prices are used as a proxy mostly, negatively affect food prices. For this reason, a positive (increasing) relationship between energy prices and global food prices is expected. Moreover, Brent crude oil prices are used as the energy price indicator.

Fertilizer prices are also included as a factor for the progress of food prices as in line with [21,22,23,24,25]. They determine that a change in fertilizer prices can cause a change in food prices. By considering these studies, it is expected that food prices increase while fertilizer prices increase. World Bank (WB) Fertilizer Price Index is used as the fertilizer price indicator.

Moreover, weather (e.g., temperature) changes can have an effect on food prices as a challenging factor. The literature that includes temperature has been increasing. For example, Refs. [7,26,27,28,29,30,31] use climate and weather changes as a factor in their studies for the case of Australia, Ethiopia, Tanzania, etc. They state that there is a high dependency between weather changes and agricultural product prices. Hence, it is expected that food prices increase while weather and climate conditions become worst. Moreover, a change in weather temperature is used as a weather indicator.

Additionally, the effects of other global factors on food prices are considered. In this context, considering [7,14,32,33], raw material prices, metals, and mineral prices, and volatility are included in the empirical analysis. That is why it can be expected that food prices increase while raw material prices increase, metals and mineral prices increase, and volatility in markets is high. As an indicator, WB Raw Material Price Index for raw material prices, WB Metals and Mineral Price Index for metals and mineral prices, and Chicago Board Options Exchange (CBOE) Volatility Index for volatility is used.

Moreover, lagged global food prices are used to take the impacts of price inertia (persistence) into account. The lagged value of the global food prices represents price inertia (persistence) and inertia could capture expectations in the agricultural markets [7,34]. The inertia could be due to expectations about global food prices driven by large agricultural shocks such as high temperatures or pandemics [35,36]. In the extant literature, [35,37,38,39,40] argue that price inertia is one of the main driving forces on the food prices for Ethiopia, Pakistan, Ethiopia and Kenya, Nigeria, and China, respectively. In line with the results of these studies, apart from [41], one-month-lagged global food prices are used as an explanatory variable by considering that global food prices are announced monthly.

Numerous researchers who study food prices in the literature consider a variety of factors in their studies. The more recent studies employ techniques such as ARDL, causality and cointegration (Granger, Toda-Yamamoto), DCC, Generalized Autoregressive Conditional Heteroscedastic (GARCH), Generalized Method of Moments (GMM), NARDL, Panel Data Analysis, Regression (Ordinary Least Squares (OLS), pooled-OLS), VAR, and Vector Error Correction Model (VECM). However, studies that compare the estimation performance of econometric models and machine learning algorithms are quite rare and this study fills this gap and accordingly contributes to the existing literature. Therefore, this study makes a specific type of contribution to the literature based on the methodology. Hence, the results of the study can present significant implications for countries in the management of food prices.

## 3. Methods

### 3.1. Research Objectives

This study aims to (i) uncover the impacts of the global factors on global food prices; (ii) determine the order of importance of the effective variables on global food prices; (iii) examine the inertia effect on global food prices; (iv) compare the empirical performances of machine learning algorithms and time series econometric models; (v) identify the best estimation model for global food prices; and (vi) develop policy proposals by benefiting from the results of the best model.

### 3.2. Data

The global food prices, economic policy uncertainty index, volatility index, and oil prices are gathered from [9]. The metals and mineral price index, raw material price index, and fertilizer price index are collected from [42]. The temperature is taken from [43].

The temperature data represent a detailed summary of the land-surface average results produced by the Berkeley Averaging method. Temperatures are in Celsius; as Earth’s land is not distributed symmetrically about the equator, there exists a mean seasonality to the global land average. For each month, the estimated land-surface average for that month and its uncertainty are reported [43].

In addition, the global food price index is composed of five commodity groups (95 price quotations within 24 commodities) and it is calculated by the weighted average of export shares of each of the groups. These commodity groups and weights are cereals (29%), oils (17%), sugar (7%), meat (33%), and dairy (14%) [44].

According to the empirical analysis, monthly frequency is used because global food prices are announced monthly. Moreover, the study covers the period between January 1991 and May 2021. This period is selected to include a large period. In this study, eight explanatory variables are included. Although including the COVID-19 pandemic as a variable was going to be planned, unfortunately, it could not be realized because the pandemic has been seen since March 2020 officially, and there are not enough observations for a monthly basis dataset. Therefore, although time intervals regarding the pandemic are included, the pandemic cannot be included as a variable. Table 1 presents a summary of the variables that are included in the analysis.

### 3.3. Methodology

**Figure 1:**
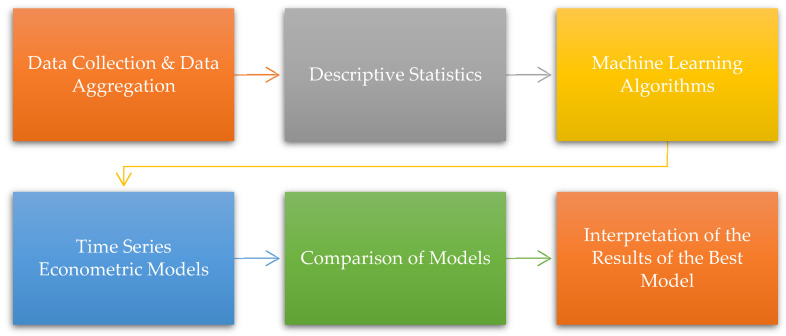
The proposed empirical model.

The proposed methodology, which has six steps, is shown in Figure 1. In this context, the following methodology is applied:

▪ Step 1: The large period is selected for data collection. Monthly data covering January 1991–May 2021 period, which has 365 observations, are used.
▪ Step 2: The descriptive statistics for each variable such as mean, standard deviation, coefficient of variation, minimum, maximum, etc. are calculated.
▪ Step 3: The dataset that includes 365 monthly observations is divided into two sub-periods: (i) the training (first 80% observations) and (ii) the testing dataset (last 20% observations), firstly. Hence, the training dataset consists of 292 observations from January 1991 to April 2015, the testing dataset contains 73 observations from May 2015 to May 2021. The training period is known as the model construction period and the testing period is known as the model validation period. After this, selected machine learning algorithms are performed. In this context, Multi-layer Perceptron (MLP), Multivariate Adaptive Regression Splines (MARS), Support Vector machines (SVM), Random Forest (RF), Extreme Gradient Boosting (XGB), and k-Nearest Neighbors (kNN) algorithms are included.
▪ Step 4: Selected time series econometrics models are applied. In line with this, Threshold regression, Markov Switching Regression, OLS, Dynamic OLS (DOLS), and Fully Modified OLS (FMOLS) are covered.
▪ Step 5: The results of the machine learning algorithms and time series econometric models are compared according to the goodness of fit criteria. In this context, Root Mean Squared Error (RMSE), Mean Absolute Error (MAE), and *R*^2^ are considered.

(1) $$\mathrm{RMSE}=\sqrt{\frac{\sum {\left(y_{i}-{\hat{y}}_{i}\right)}^{2}}{n}}$$

(2) $$\mathrm{MAE}=\frac{\left|\left(y_{i}-{\hat{y}}_{i}\right)\right|}{n}$$

(3) $$R^{2}=1-\frac{\sum {\left(y_{i}-{\hat{y}}_{i}\right)}^{2}}{\sum {\left(y_{i}-\bar{y}\right)}^{2}}$$

where $y_{i}$ is the actual value, ${\hat{y}}_{i}$ is the predicted value from the model, $\bar{y}$ is the mean of actual values and n is the number of observations. These metrics, RMSE and MAE, are used to diagnose the variation in the error and measure the accuracy of the variable. *R*^2^ measures the amount of variation that can be explained by the model [45].

▪ Step 6: The best model is selected from alternative models according to the goodness of fit criteria and its results are interpreted.

Both machine learning algorithms and time series econometric models applied in the empirical analysis are not explained in detail to avoid extending the article so much for lack of space. More information can be obtained from [46,47,48] for time series econometric models and [49,50,51] for machine learning algorithms.

## 4. Empirical Analysis

### 4.1. Descriptive Statistics

**Figure 2:**
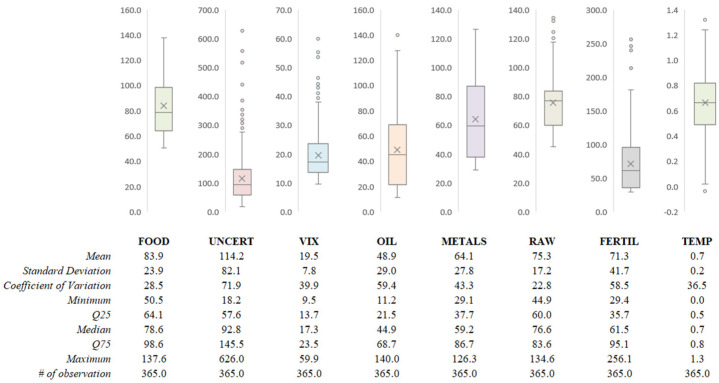
Descriptive statistics.

One of the most beneficial methods to understand the data distribution is to examine the Box and Whisker Plot alongside analyzing the descriptive statistics. The Box and Whisker plot and descriptive statistics of each variable are presented in Figure 2.

Based on Figure 2, almost all variables have right-skewed distributions and have several outliers, except food price and temperature. The coefficient of variation values of UNCERT, OIL, and FERTIL are relatively higher than others. Moreover, FOOD and RAW have relatively lower coefficients of variation among others. These results indicate that the volatility of UNCERT, OIL, and FERTIL is relatively high, while the volatility of FOOD and RAW is low.

### 4.2. Machine Learning Algorithms versus Time Series Econometric Models

In the study, six different machine learning algorithms and five different time series econometric models are estimated to determine the best model. Thus, the best model, which has the highest accuracy and the lowest error statistics, is determined by comparing the estimation performance of alternative machine learning algorithms and time series econometric models. The comparison of performance statistics by models is presented in Table 2.

**Figure 3:**
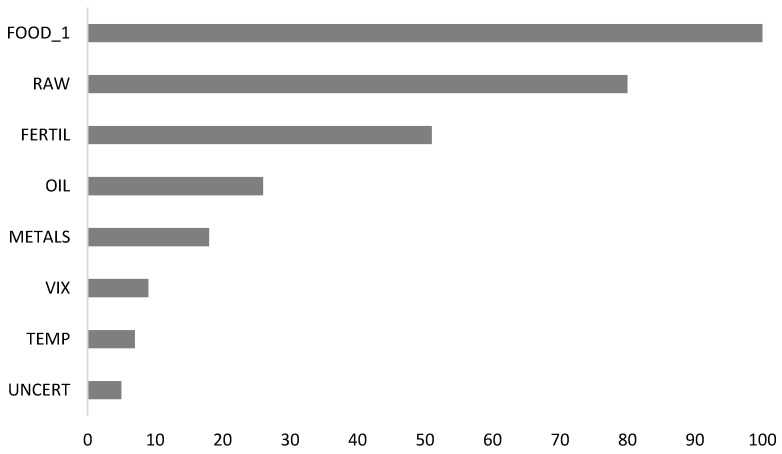
Variable importance analysis.

As seen in Table 2, it is revealed that machine learning algorithms outperform time series econometric models according to the goodness of fit criteria, which include RMSE, MAE, and *R*^2^. In machine learning algorithms, the MLP algorithm produces the best estimation results in both training and testing samples among others. In addition, the overfitting problem is also double-checked in the study based on the results given in Table 2 since the machine learning algorithms tend to overfit. In the literature, it is suggested that splitting data into training and testing parts is one of the basic ways to detect the overfitting problem. Overfitting occurs if the model performance statistics in the training test are significantly better than the test set [52]. In this study, it is seen that there is no significant gap between the training and testing sets in terms of goodness of fit criteria. Thus, it can be stated that there is no overfitting problem for machine learning algorithms. After determining the best model, variable importance analysis, which is based on the MLP algorithm, is evaluated. The result of the variable importance analysis is displayed in Figure 3.

**Figure 4:**
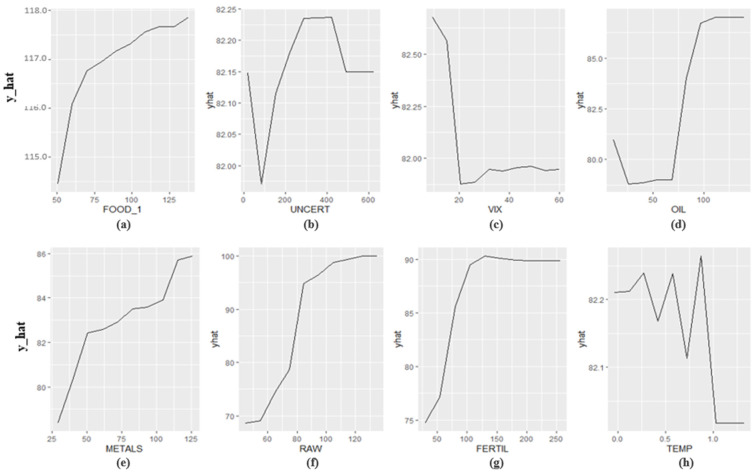
Critical thresholds of the independent variables. (**a**) FOOD_1, (**b**) UNCERT, (**c**) VIX, (**d**) OIL, (**e**) METALS, (**f**) RAW, (**g**) FERTIL and (**h**) TEMP.

In Figure 3, the most influential variable is FOOD_1 (i.e., the one-month lag of the dependent variable) followed by RAW, FERTIL, OIL, and METALS, respectively. The relative importance of VIX, TEMP, and UNCERT is not high with regard to the before-mentioned indicators. In addition, the critical thresholds of the variables affecting food price levels significantly are examined and the critical thresholds are shown in Figure 4.

There is a positive correlation between FOOD and FOOD_1, UNCERT, OIL, METALS, RAW, and FERTIL. This means that food price increases as FOOD_1, UNCERT, OIL, METALS, RAW, and FERTIL increase. Moreover, it is revealed that food prices decrease as VIX is less than 20. Furthermore, the critical thresholds for FOOD_1, UNCERT, VIX, OIL, METALS, RAW, FERTIL, and TEMP are 75, 200, 20, 75, 50, 80, 50, and 0.8, respectively. In other words, if UNCERT is higher than 200 or OIL is higher than 75 or METALS is higher than 50 or RAW is higher than 80, or FERTIL is higher than 50, global food prices will be at a relatively higher level.

**Figure 5:**
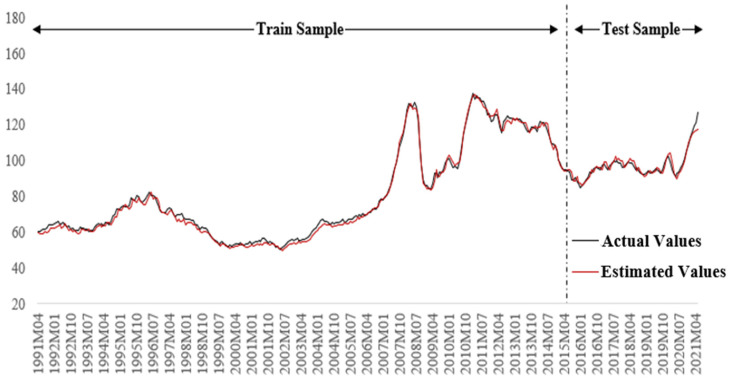
Actual and estimated values of the global food prices.

Finally, actual versus estimated values obtained from the MLP algorithm is given in Figure 5 to double-check the model results visually. Generally, actual and estimated values should be too close for an acceptable model.

As can be seen in Figure 5, actual and estimated values are very close to each other, even during the time of sudden increases or decreases, which reveals that the MLP algorithm can successfully estimate global food prices with the independent variables considered in both training and testing periods.

### 4.3. Discussion and Policy Implications

With the application of the proposed empirical model, it is determined that machine learning algorithms provide much superior estimation performance than time series econometric models. It is found that the MLP is the best estimation method for global food prices based on performance metrics. For this reason, the impacts of global factors on global food prices are examined in detail by using the MLP algorithm.

The MLP outcomes present that one-month-lagged global food prices are the most significant factor followed by raw material prices, fertilizer prices, crude oil prices, metals and mineral prices, volatility index, temperature changes, and economic policy uncertainty, respectively. Generally, global factors included in the empirical analysis have a positive relationship with global food prices. On the other hand, volatility and temperature have a changing (i.e., both increasing and decreasing) impact on global food prices up to certain levels. Moreover, although other factors have a positive relationship, the degree of impact can change according to thresholds. These results show that thresholds of the global variables should be also considered in policy development and implementation processes.

The MLP results validate the hypotheses that are the research objectives of the study: (i) an increase in the explanatory variables generally causes an increase in global food prices; (ii) the impacts of the global explanatory variables change according to the thresholds; (iii) one-month-lagged global food prices are found to be the most important variable, which indicates price inertia; (iv) machine learning algorithms have a higher estimation performance than time series econometric models.

The results that are obtained from the MLP algorithm are generally consistent with [12,13] for the economic uncertainty; [14,32] for the metals and mineral prices; [19,20] for the oil prices; [24,25] for the fertilizer prices; [37,38,40] for the lagged global food prices as indicating the importance of the inertia for agricultural prices. On the other hand, although it was expected that volatility and temperature increases would have an adverse effect [29,30,31], the results do not support this expectation. Increasing volatility causes a decrease in food prices up to a certain level, and it causes an increase after this level. A similar condition is also valid for the impact of temperature changes. Nevertheless, it can be generally concluded that global variables are significant for global food prices and an increase in global factors causes generally an increase in global food prices as well.

This study provides multiple policy implications for countries to better analyze global food prices. First, the most significant explanatory variable, lagged food prices, indicates the importance of inertia in the agricultural markets. The results reveal that analyzing inertia dynamics is important to keep food prices under control and achieve stability. From this perspective, country-based analyses should be made by considering country-based differences. Hence, country-specific causes of food price inertia can be defined.

Second, raw material prices should be focused on as the second most influential variable. In this context, solving problems in the supply chain, especially in main raw materials, such as timber, cotton, natural rubber, and tobacco, is crucial. The production of more raw materials or the usage of current raw materials in a more efficient way can be beneficial in terms of decreasing or limiting price increases in the food prices caused by raw material prices increase.

Third, fertilizer prices should be handled carefully. In this regard, it can be proposed that raw material costs, which are used in the manufacture of chemical fertilizers such as ammonia and natural gas, should be decreased. To do so, some incentives can be provided. In addition, increasing and disseminating usage of natural fertilizers can be evaluated within the scope of transformation to a green world. Moreover, the production of fertilizer either chemical or organic, which does not exist currently, can be initialized and this may contribute to the decrease of total global demand transportation cost of fertilizer. Hence, the demand for chemical fertilizer decreases while the supply of total fertilizer increases, which may result in a decrease in fertilizer prices and food prices in turn.

Fourth, oil prices should be managed critically. Energy prices are an important cost item for food production because of usage in almost all steps. Thus, using oil more efficiently would be very helpful. More importantly, the usage of more renewable energy sources, such as solar, wind, and hydroelectricity, can be the right solution to decrease the negative impacts of energy (oil) prices on food prices. In this framework, providing some incentives and making legislation to accelerate renewable energy investments should be taken into consideration.

Fifth, metals and mineral prices should also be focused on. Especially, some metals and minerals, such as aluminum, copper, iron, nickel, and tin, are critically important for food prices. Similar to raw material prices and fertilizer prices, increasing the production of such metals can be an option to limit the negative effects on food prices. Another option can be that alternative products in some processes, such as packing, can be used and such a shift can decrease the demand for metals and minerals. Hence, relative relief can be achieved in terms of the impact of metals and mineral prices on food prices.

Sixth, volatility and economic policy uncertainty should be underlined. Although volatility and economic policy uncertainty at the global level can be affected by a variety of issues, they can be limited or stabilized by decreasing political conflicts, keeping the COVID-19 pandemic under control, ending the trade war, and increasing collaboration among countries rather than increasing conflicts.

Seventh, weather (temperature) changes should be considered. It is a well-known fact that there has been a global warming trend and some efforts, such as climate agreements, exist. Thus, the participation of all countries in climate change agreements, such as the Kyoto Protocol (expired) and Paris (in progress), is highly recommended. In addition, the implementation of such agreements’ requirements is significant and this can contribute positively to limiting greenhouse emissions on a global basis. In stabilizing global warming, adverse effects that result from this area can be eliminated.

Eighth, there are some thresholds for the explanatory variables. These thresholds show that the development and timely implementation of policies by considering the global economic environment can contribute to achieving stability in global food prices by stabilizing the negative impacts of the explanatory variables.

Ninth, machine learning algorithms (i.e., the MLP algorithm in this study) provide better estimation performances than time series econometric models. Therefore, policymakers can benefit from such approaches to improve their estimation to develop and implement better policies.

Lastly, handling global food prices as a macro-prudential concern at the global level is highly recommended because the progress of food prices adversely affects all countries and people. Hence, global variables, confirmed as efficient and meaningful in the study, should be taken into account, which can help policymakers in their policy development and implementation processes through which countries can benefit from the stability of food prices.

## 5. Conclusions

This study investigates the effects of global factors on global food prices by comparing estimation performances of alternative machine learning algorithms and time series econometric models with comprehensive econometric and statistical approaches. The empirical analysis results reveal that machine learning algorithms present better estimation performance than the time series econometric models. Among the algorithms, the MLP algorithm is determined as the best estimation model. The findings of the MLP algorithm show that one-month-lagged global food prices are the most significant variable followed by raw material prices, fertilizer prices, and oil prices, respectively. The results obtained from the MLP algorithm are found to be largely consistent with the literature, which confirms that the result is robust based on the comparison of actual and estimated values of global food prices.

The empirical results highlight the effects of fluctuations of the global variables on global food prices and reveal the higher estimation performance of machine learning algorithms for global food prices. For future research, the analyses can be extended by using high-frequency data, which is not available to the public, to illuminate the path for a more stable food price market at the global level. Moreover, the COVID-19 pandemic, which is thought of as one of the reasons behind the increase in food prices, can be included in future studies. In this study, the COVID-19 pandemic cannot be included due to the lack of enough data accumulation. It may be beneficial for future studies to repeat this analysis in the post-pandemic period by including the pandemic as a variable. Moreover, new machine learning algorithms and time series econometric models that are not included in this study can be used in forthcoming studies. In addition, forthcoming studies can also consider including rational expectations about weather shocks, which are related to the temperature variable in this study, in future analysis [31]. In addition, the food price index is an aggregation of regional food prices and the price variation can be differentiated across regions. For this reason, new methods can be tried in future studies that can include inter-regional price differences in the model. Moreover, this study considers only supply-side factors, which is an important limitation. The studies can include both demand and supply side factors to examine the development of global food prices. Furthermore, by considering that this study uses oil prices and weather temperature as the proxies of energy prices and weather changes, new studies can also use other types of energy prices (i.e., natural gas, coal, renewable, nuclear) and precipitation as the proxy of these indicators. Hence, new insights could be obtained from these perspectives. Forthcoming studies can even use adaptative expectations for price inertia instead of lagged global food prices [41].

Implementing various econometric and statistical models may offer different and more opportunities to obtain different and more insights regarding global food prices so that more comprehensive policies can be developed. Last but not least, the lagged food price variable was found to be the most important independent variable, which indicates the importance of inertia in agricultural prices. This result highlights the importance of country-specific studies, which focus on inertia dynamics. In addition to the explanatory global variables used in the study, country-based national factors either macroeconomic or financial variables can be included in future studies that may investigate national food prices. For the time being, this is not in the scope of this study. It is expected that countries that have been facing increasing domestic food prices can benefit from the findings of this study by considering global and national factors at the same time.

## Figures and Tables

**Table 1:** The definitions of the variables.

Variables	Symbol	Description	Expected Nexus	Sources
Global Food Prices *	FOOD	United Nations Food and Agriculture World Food Price Index	N/A	[9]
Lagged Food Prices	FOOD_1	One-Month Lagged Global Food Prices	+	[9]
Economic Policy Uncertainty	UNCERT	US Economic Policy Uncertainty Index	+	[9]
Volatility Index	VIX	CBOE Volatility Index	+	[9]
Oil Prices	OIL	Brent Crude Oil Price (USD per Barrel)	+	[9]
Metals and Mineral	METALS	Metals and Mineral Price Index	+	[42]
Raw Material Price	RAW	Raw Material Price Index	+	[42]
Fertilizer Price	FERTIL	Fertilizer Price Index	+	[42]
Temperature	TEMP	Weather Temperature Changes	+	[43]

* denotes the dependent variable.

**Table 2:** Model comparison statistics.

Groups	Models	Train			Test		
		RMSE	MAE	*R* ^2^	RMSE	MAE	*R* ^2^
Time Series Econometric Models	Threshold	10.519	8.921	0.933	10.506	9.146	0.958
	Markov	20.956	19.694	0.887	8.782	7.072	0.945
	DOLS	15.301	13.063	0.900	9.498	7.011	0.936
	OLS	15.041	13.358	0.856	10.516	8.157	0.924
	FMOLS	14.684	12.544	0.851	9.988	7.516	0.923
Machine Learning Algorithms	MLP	0.087	0.064	0.994	1.900	1.479	0.943
	MARS	2.107	1.481	0.993	1.910	1.501	0.939
	SVM	2.196	1.585	0.993	1.991	1.566	0.929
	RF	2.681	1.748	0.989	2.262	1.690	0.909
	XGB	2.826	1.945	0.988	3.305	2.501	0.846
	kNN	4.963	3.493	0.962	5.238	3.715	0.637

## Data Availability

The data are avaliable from the corresponding author.
